# Supplementary material for: Resolving atomic SAPO-34/18 intergrowth architectures for methanol conversion by identifying light atoms and bonds
Source: Nat Commun. 2021 Apr 13;12:2212. doi: 10.1038/s41467-021-22438-z (PMC8044160; doi:10.1038/s41467-021-22438-z)
Supplement: Supplementary file 1 — Supplementary Information [file 41467_2021_22438_MOESM1_ESM.pdf]

# Supplementary Information

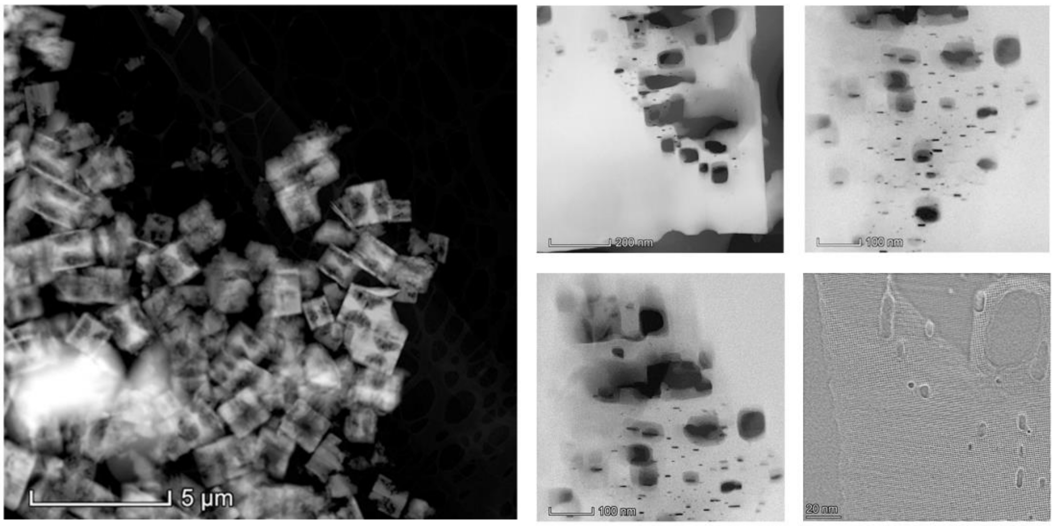


Supplementary Figure 1. STEM images of the hierarchical catalysts. The mesopores, macropores and hollow areas inside the crystals are clearly imaged.

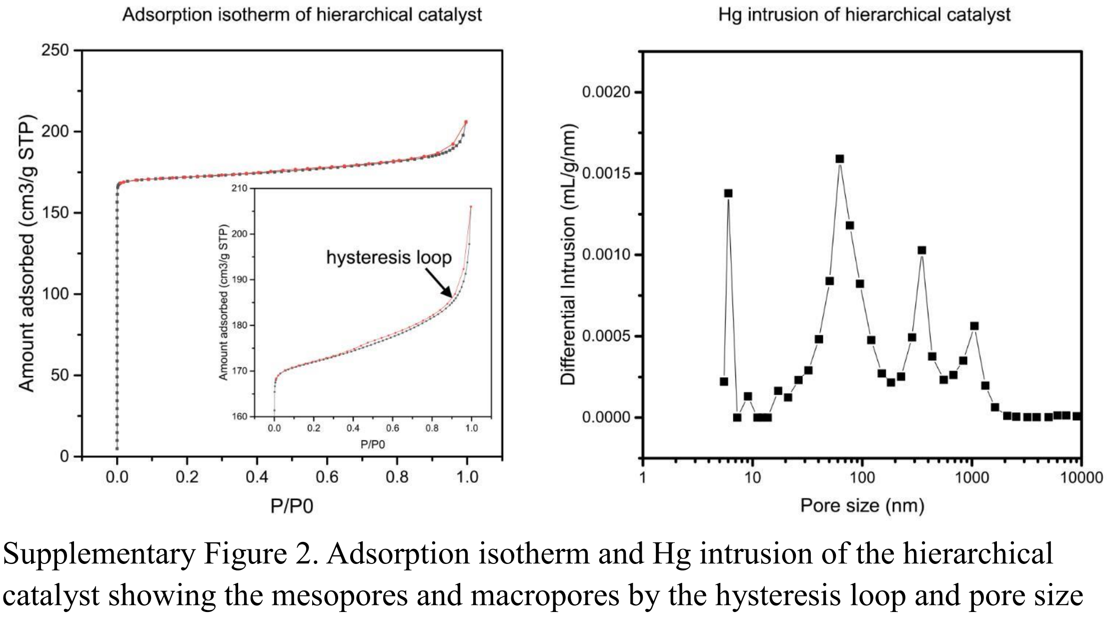


Supplementary Figure 2. Adsorption isotherm and Hg intrusion of the hierarchical catalyst showing the mesopores and macropores by the hysteresis loop and pore size distribution.

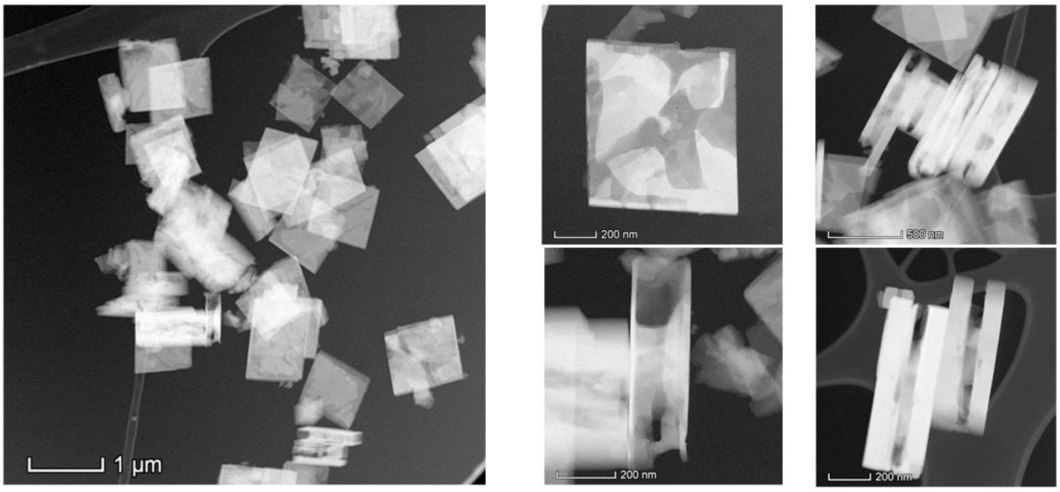


Supplementary Figure 3. STEM images of the sandwich catalysts. The thickness of lamellas can be measured as 10-100 nm.

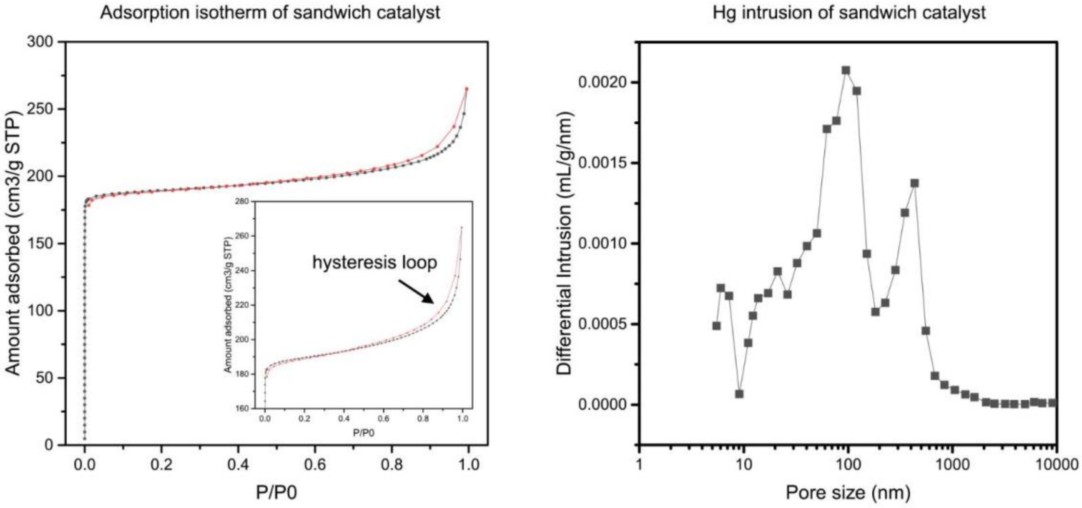


Supplementary Figure 4. Adsorption isotherm and Hg intrusion of the sandwich catalyst showing the mesopores and macropores by the hysteresis loop and pore size distribution.

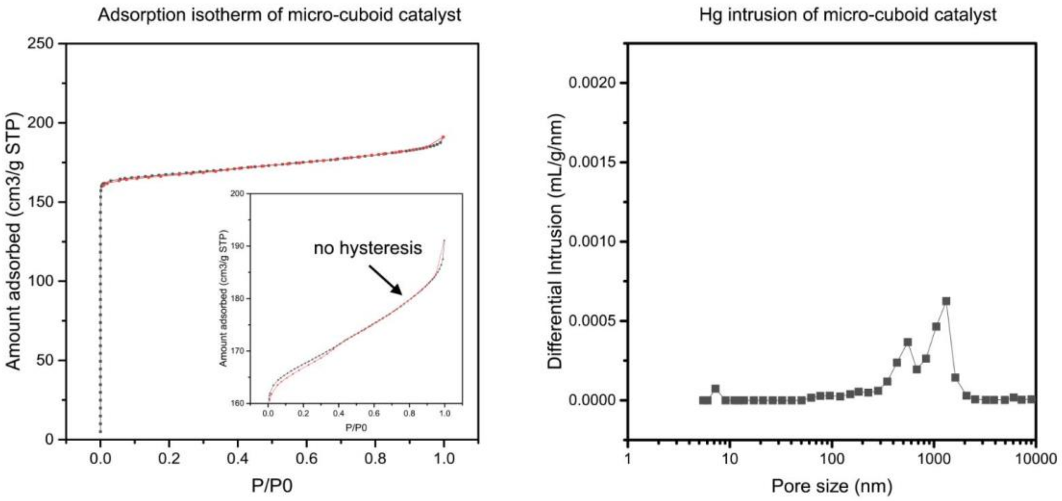


Supplementary Figure 5. Adsorption isotherm and Hg intrusion of the micron-sized cuboid catalyst. There is nearly no mesopore and macropore that can be detected in this catalyst.

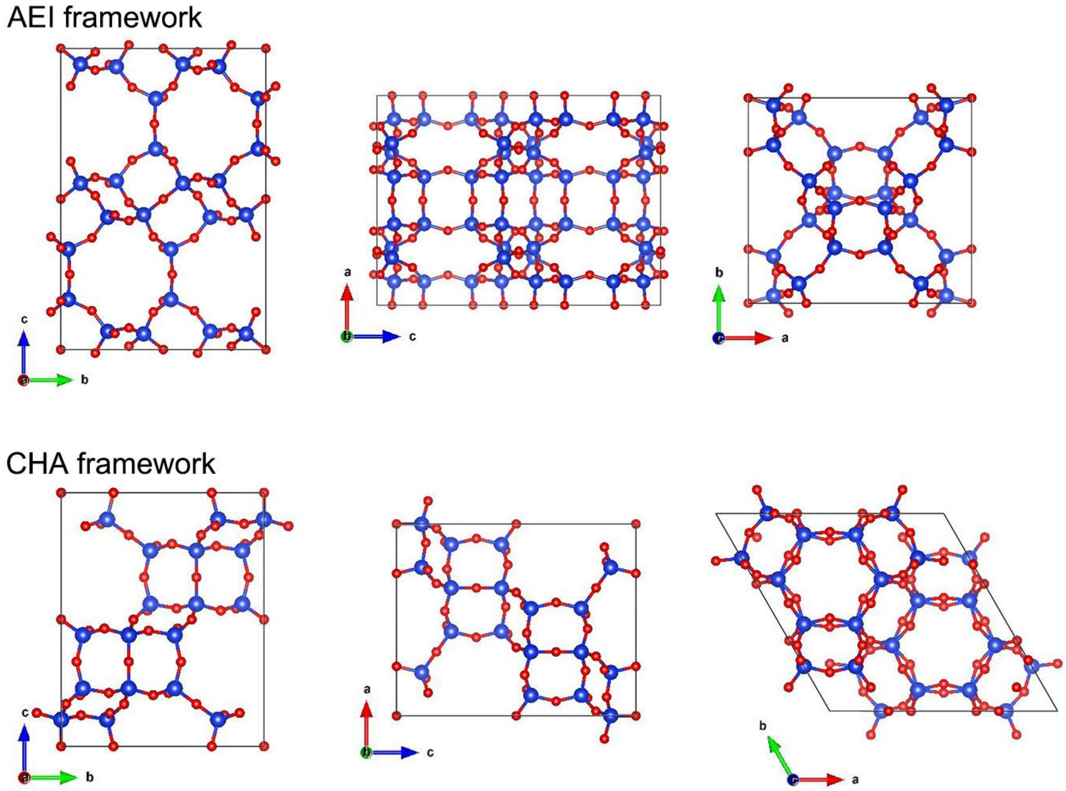


Supplementary Figure 6. Atomic models of AEI (SAPO-18) and CHA (SAPO-34) frameworks.

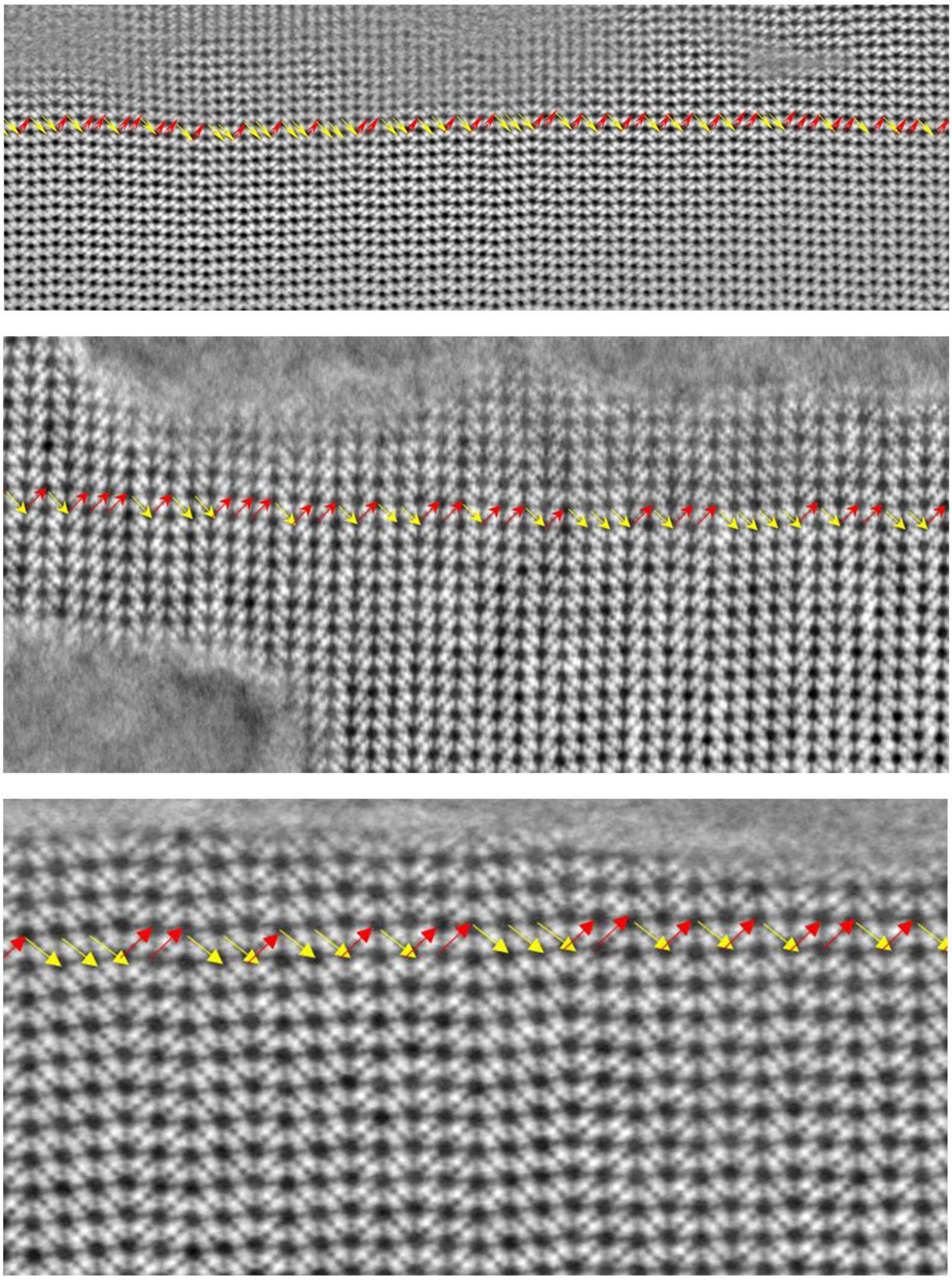

Supplementary Figure 7. More iDPC-STEM images showing the distribution and stacking sequences of SAPO-34 and SAPO-18 in the hierarchical catalysts.

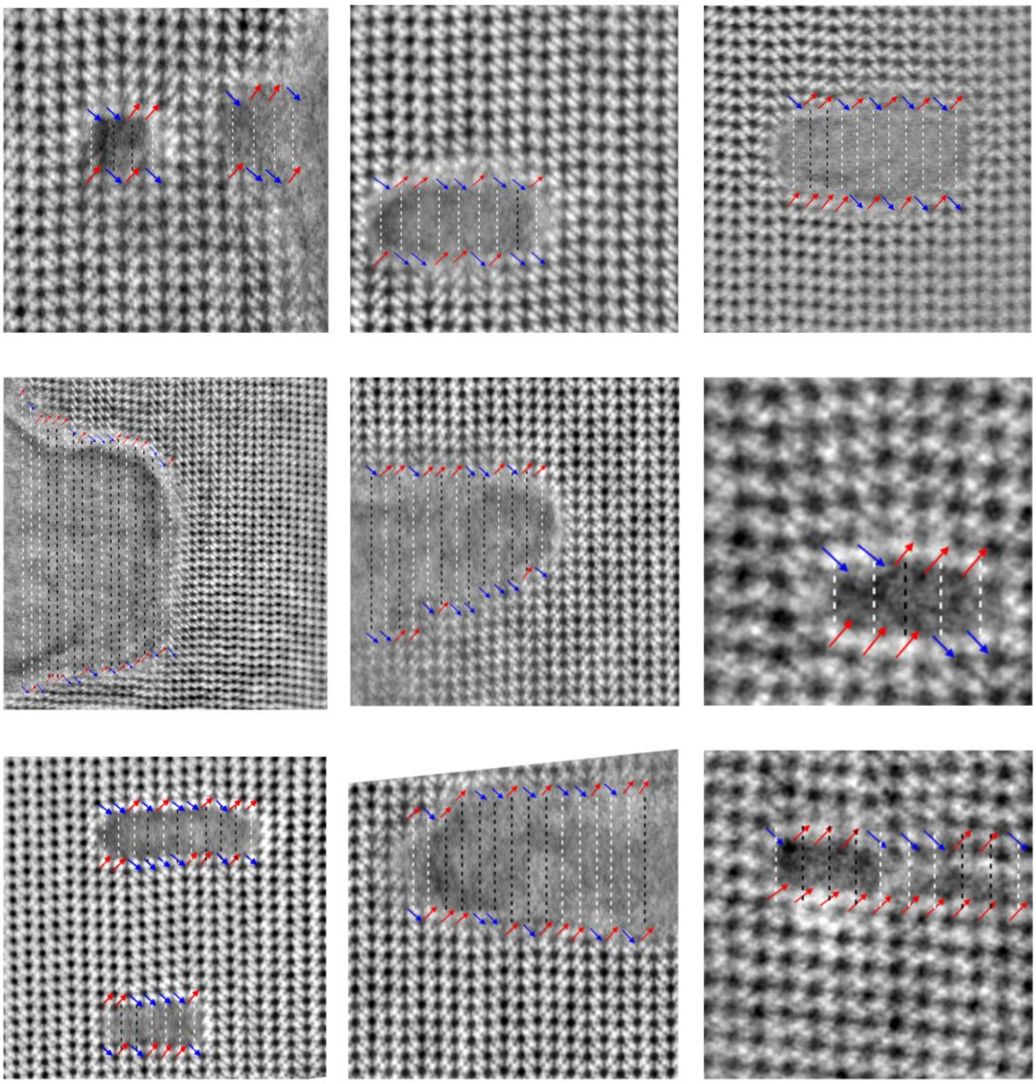

Supplementary Figure 8. More iDPC-STEM images showing the mismatched lattices surrounding the through mesopores in SAPO-34/18 intergrowths. These images confirm the correlation between the mesopore formation and lattice mismatching.

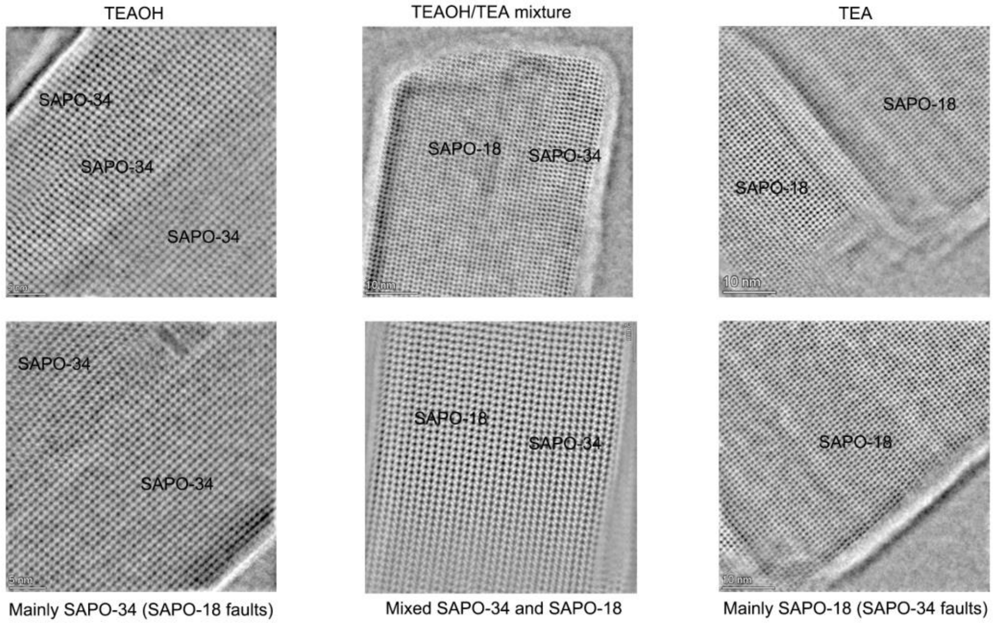


Supplementary Figure 9. IDPC-STEM images of the samples directed by pure TEAOH, TEAOH/TEA mixture (TEA:TEAOH=3.43) and pure TEA respectively. The corresponding PXRD results are given in Fig. 4b. At this Si content in feed, the TEAOH-template sample is dominant by SAPO-34, while the TEA-template sample is dominant by SAPO-18. The sample directed by dual TEAOH/TEA template is a mixture of SAPO-34 and SAPO-18 domains.

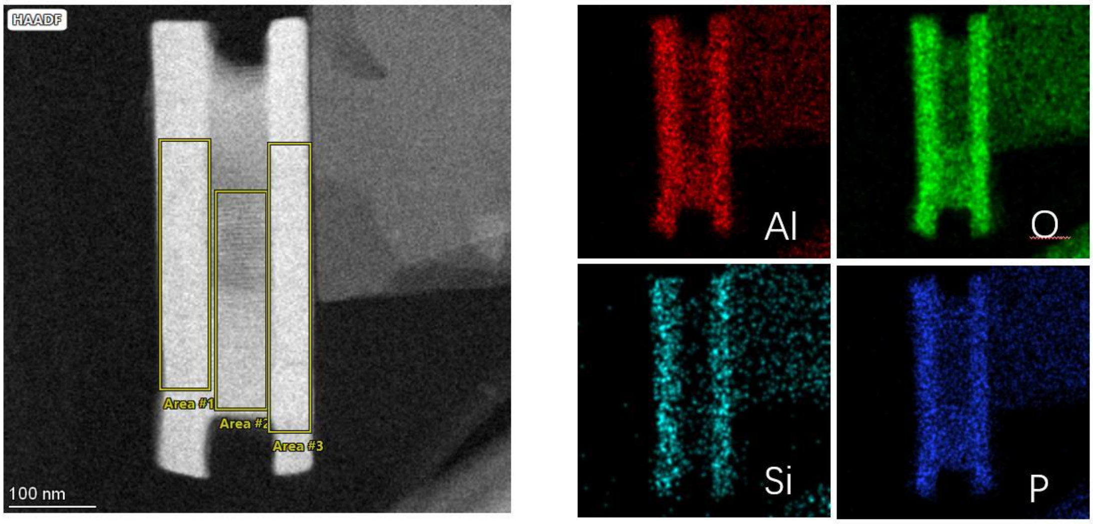


Supplementary Figure 10. EDS mapping of the sandwich SAPO-34/18 crystal studied in Fig. 3 and 4. The proportions of different elements in three areas are given in Supplementary Table 1.

Supplementary Table 1. The proportions of different elements in three areas in Supplementary Figure 10.

| | Element | Atomic Fraction (%) | Atomic Error (%) | Mass Fraction (%) | Mass Error (%) |
|---|---|---|---|---|---|
| **Area1** | O | 66.2 | 6.42 | 52.22 | 3.21 |
| | Al | 17.49 | 3.85 | 23.26 | 4.82 |
| | Si | 2.75 | 0.6 | 3.81 | 0.78 |
| | P | 13.56 | 2.89 | 20.71 | 4.14 |
| **Area2** | O | 65.56 | 6.84 | 51.36 | 3.39 |
| | Al | 17.95 | 4.01 | 23.71 | 4.94 |
| | Si | 0.62 | 0.16 | 0.86 | 0.2 |
| | P | 15.87 | 3.44 | 24.07 | 4.85 |
| **Area3** | O | 65.91 | 6.43 | 51.88 | 3.2 |
| | Al | 17.55 | 3.87 | 23.3 | 4.82 |
| | Si | 2.66 | 0.58 | 3.68 | 0.75 |
| | P | 13.88 | 2.97 | 21.15 | 4.23 |

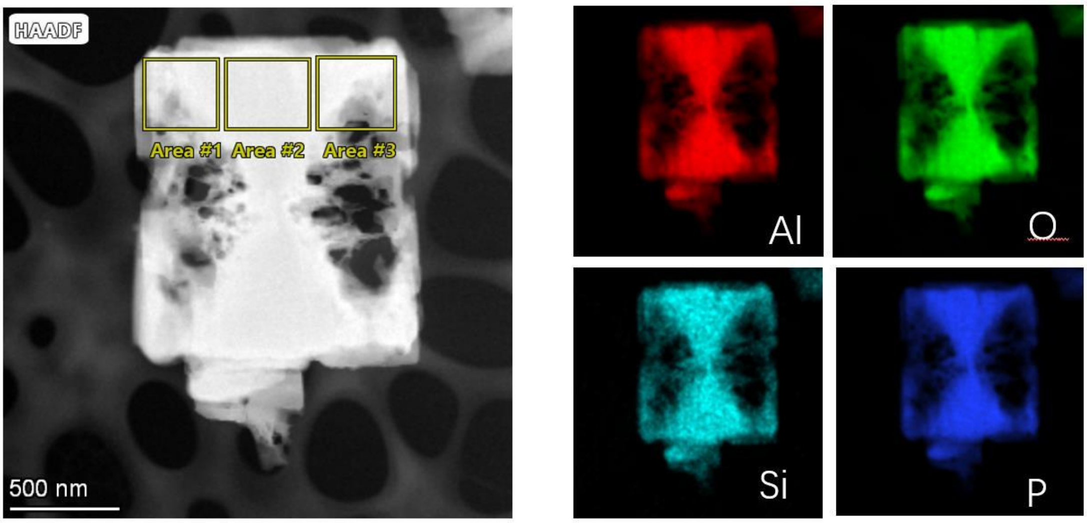


Supplementary Figure 11. EDS mapping of the hierarchical SAPO-34/18 crystal studied in Fig. 3 and 4. The proportions of different elements in three areas are given in Supplementary Table 2.

Supplementary Table 2. The proportions of different elements in three areas in Supplementary Figure 11.

| | Element | Atomic Fraction (%) | Atomic Error (%) | Mass Fraction (%) | Mass Error (%) |
|---|---|---|---|---|---|
| **Area1** | O | 69.35 | 6.32 | 55.75 | 3.23 |
| | Al | 15.96 | 3.47 | 21.64 | 4.46 |
| | Si | 1.71 | 0.37 | 2.41 | 0.49 |
| | P | 12.98 | 2.74 | 20.2 | 4.01 |
| **Area2** | O | 68.07 | 6.51 | 54.26 | 3.32 |
| | Al | 16.51 | 3.62 | 22.19 | 4.58 |
| | Si | 1.69 | 0.36 | 2.36 | 0.48 |
| | P | 13.73 | 2.92 | 21.18 | 4.22 |
| **Area3** | O | 68.64 | 6.39 | 54.93 | 3.24 |
| | Al | 16.28 | 3.58 | 21.97 | 4.56 |
| | Si | 1.73 | 0.38 | 2.42 | 0.5 |
| | P | 13.35 | 2.83 | 20.68 | 4.12 |

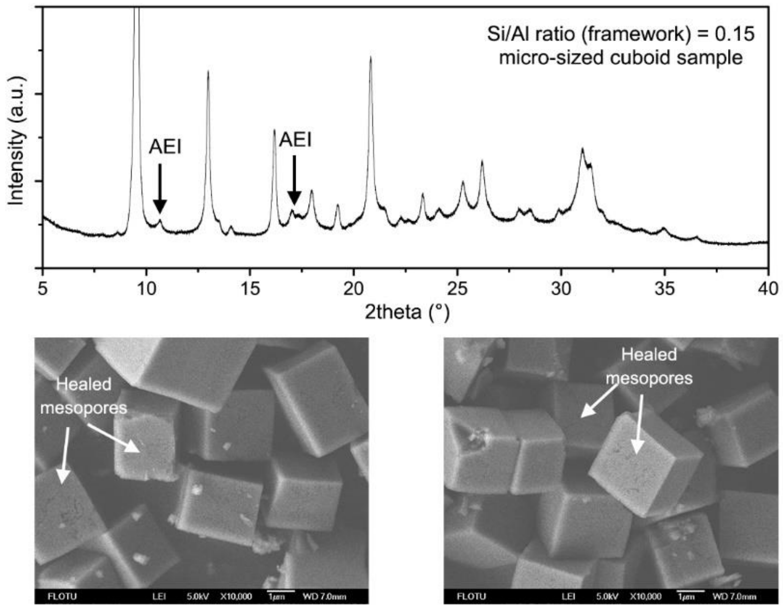


Supplementary Figure 12. PXRD result and SEM images of micro-sized cuboid sample that we used in Fig. 5 as a comparison group. This cuboid sample was synthesized with a low Si content but a higher temperature, where the mesopores can be healed to form nearly solid cuboids.

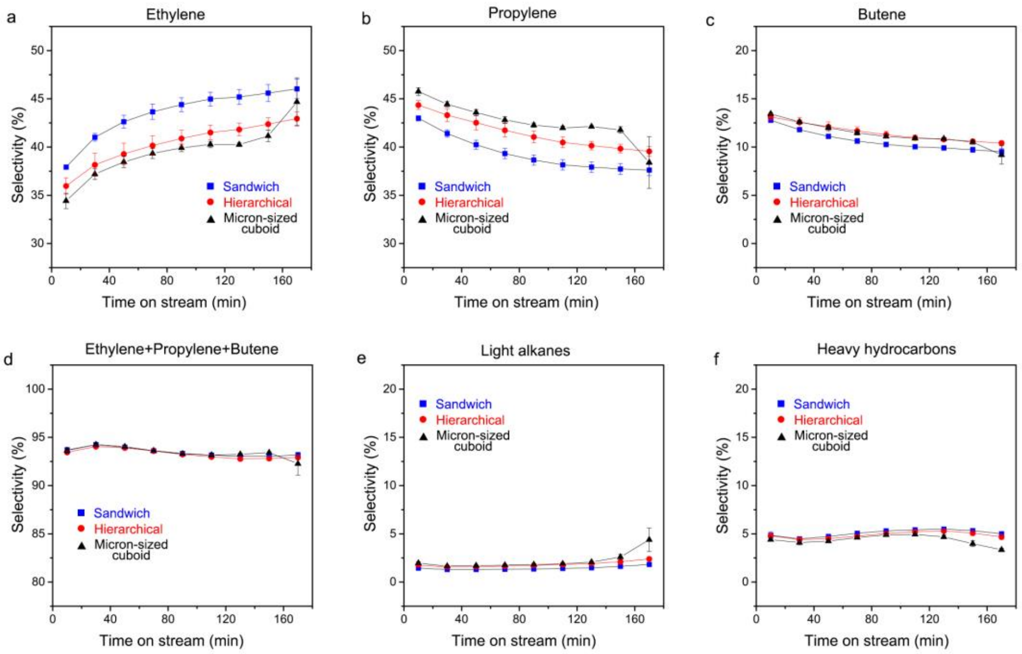


Supplementary Figure 13. (**a**) Gas selectivity of ethylene in methanol conversion. (**b**) Gas selectivity of propylene. (**c**) Gas selectivity of butene. (**d**) Gas selectivity of light olefins ($C_2$-$C_4$ olefins). (**e**) Gas selectivity of light alkanes ($C_1$-$C_4$ alkanes). (**f**) Gas selectivity of heavy hydrocarbons ($C_5$-$C_7$). These catalysts show very high gas selectivity of light olefins ($C_2$-$C_4$ olefins, ~93%), while very few alkanes ($C_1$-$C_4$ alkanes, ~2%) and heavy hydrocarbons ($C_5$-$C_7$, ~5%) were produced. The error bars represent the standard deviations of four sets of data in repeated experiments.
